# Genetic Association Study Identifies *HSPB7* as a Risk Gene for Idiopathic Dilated Cardiomyopathy

**DOI:** 10.1371/journal.pgen.1001167

**Published:** 2010-10-21

**Authors:** Klaus Stark, Ulrike B. Esslinger, Wibke Reinhard, George Petrov, Thomas Winkler, Michel Komajda, Richard Isnard, Philippe Charron, Eric Villard, François Cambien, Laurence Tiret, Marie-Claude Aumont, Olivier Dubourg, Jean-Noël Trochu, Laurent Fauchier, Pascal DeGroote, Anette Richter, Bernhard Maisch, Thomas Wichter, Christa Zollbrecht, Martina Grassl, Heribert Schunkert, Patrick Linsel-Nitschke, Jeanette Erdmann, Jens Baumert, Thomas Illig, Norman Klopp, H.-Erich Wichmann, Christa Meisinger, Wolfgang Koenig, Peter Lichtner, Thomas Meitinger, Arne Schillert, Inke R. König, Roland Hetzer, Iris M. Heid, Vera Regitz-Zagrosek, Christian Hengstenberg

**Affiliations:** 1 Klinik und Poliklinik für Innere Medizin II, Universitätsklinikum Regensburg, Regensburg, Germany; 2 Institute for Gender in Medicine, Center for Cardiovascular Research, Charité Campus Mitte, Berlin, Germany; 3 Department of Cardiothoracic and Vascular Surgery, German Heart Institute Berlin, Berlin, Germany; 4 Institute of Epidemiology, Public Health, and Gender Studies, University of Regensburg, Regensburg, Germany; 5 Institut National de la Santé et de la Recherche Médicale (INSERM) UMR-S956, University Pierre et Marie Curie (Paris 6), Institut de Cardiologie, Département de Génétique, AP-HP, Hôpital Pitié-Salpêtrière, Paris, France; 6 Institut National de la Santé et de la Recherche Médicale (INSERM), UMR-S937, University Pierre et Marie Curie (Paris 6), Paris, France; 7 Service de Cardiologie, Hôpital Bichat, AP-HP, Paris, France; 8 Service de Cardiologie, Université de Versailles-Saint Quentin, Hôpital Ambroise Paré, AP-HP, Boulogne, France; 9 Service de Cardiologie, Hôpital Laennec, Nantes, France; 10 Service de Cardiologie B et Laboratoire d'Electrophysiologie Cardiaque, Pole Cœur Thorax Vasculaire Hémostase, Centre Hospitalier Universitaire Trousseau, Tours, France; 11 Service de Cardiologie, Hôpital Cardiologique, Lille, France; 12 Klinik für Innere Medizin I - Kardiologie, Philipps-Universität Marburg, Marburg, Germany; 13 Medizinische Klinik und Poliklinik C, Kardiologie und Angiologie, Universitätsklinikum Münster, Münster, Germany; 14 Medizinische Klinik II, Universität zu Lübeck, Lübeck, Germany; 15 Institute of Epidemiology, HelmholtzZentrum München, München-Neuherberg, Germany; 16 Central Hospital of Augsburg, MONICA/KORA Myocardial Infarction Registry, Augsburg, Germany; 17 Department of Internal Medicine II – Cardiology, University of Ulm Medical Center, Ulm, Germany; 18 Institute of Human Genetics, HelmholtzZentrum München, München-Neuherberg, Germany; 19 Institute of Human Genetics, Klinikum rechts der Isar, Technische Universität München, München, Germany; 20 Institute of Medical Biometry and Statistics, University of Lübeck, Lübeck, Germany; Georgia Institute of Technology, United States of America

## Abstract

Dilated cardiomyopathy (DCM) is a structural heart disease with strong genetic background. Monogenic forms of DCM are observed in families with mutations located mostly in genes encoding structural and sarcomeric proteins. However, strong evidence suggests that genetic factors also affect the susceptibility to idiopathic DCM. To identify risk alleles for non-familial forms of DCM, we carried out a case-control association study, genotyping 664 DCM cases and 1,874 population-based healthy controls from Germany using a 50K human cardiovascular disease bead chip covering more than 2,000 genes pre-selected for cardiovascular relevance. After quality control, 30,920 single nucleotide polymorphisms (SNP) were tested for association with the disease by logistic regression adjusted for gender, and results were genomic-control corrected. The analysis revealed a significant association between a SNP in *HSPB7* gene (rs1739843, minor allele frequency 39%) and idiopathic DCM (p = 1.06×10^−6^, OR = 0.67 [95% CI 0.57–0.79] for the minor allele T). Three more SNPs showed p < 2.21×10^−5^. *De novo* genotyping of these four SNPs was done in three independent case-control studies of idiopathic DCM. Association between SNP rs1739843 and DCM was significant in all replication samples: Germany (n = 564, n = 981 controls, p = 2.07×10^−3^, OR = 0.79 [95% CI 0.67–0.92]), France 1 (n = 433 cases, n = 395 controls, p = 3.73×10^−3^, OR = 0.74 [95% CI 0.60–0.91]), and France 2 (n = 249 cases, n = 380 controls, p = 2.26×10^−4^, OR = 0.63 [95% CI 0.50–0.81]). The combined analysis of all four studies including a total of n = 1,910 cases and n = 3,630 controls showed highly significant evidence for association between rs1739843 and idiopathic DCM (p = 5.28×10^−13^, OR = 0.72 [95% CI 0.65–0.78]). None of the other three SNPs showed significant results in the replication stage.

This finding of the *HSPB7* gene from a genetic search for idiopathic DCM using a large SNP panel underscores the influence of common polymorphisms on DCM susceptibility.

## Author Summary

Dilated cardiomyopathy is a severe disease of the heart muscle and often leads to chronic heart failure, eventually with the consequence of cardiac transplantation. Identification of genetic disease markers in at-risk persons could play an important role in preventive health care. Several mutations in familial forms of the disease are described. Here, we examine the role of common genetic variants on the sporadic form of dilated cardiomyopathy. By screening about 2,000 candidate genes previously related to cardiovascular disease in more than 1,900 cases and 3,600 controls, we show that a polymorphism in the *HSPB7* gene (rs1739843) is strongly associated with susceptibility to dilated cardiomyopathy. We also show that the effect on disease risk is present in both German and French cohorts. Therefore, this study is an important step towards revealing insight in the genetic background of the sporadic form of dilated cardiomyopathy.

## Introduction

Dilated cardiomyopathy (DCM) is a common form of heart muscle disease with a prevalence of 1∶2,500 in the general population. It represents a major cause of cardiovascular morbidity and mortality and is characterized by systolic dysfunction as well as dilation and impaired contraction of the ventricles, often leading to chronic heart failure and eventually requiring cardiac transplantation [1]. In about 35% of cases DCM is a familial disease [2]. However, in the sporadic form of DCM, i. e. after exclusion of affected family members and all detectable causes (also called idiopathic DCM), a genetic component is discussed, but can thus far not be assigned to single gene defects. Knowledge of genetic risk factors for both, familial and non-familial forms of DCM is important to initiate treatment prior to symptomatic onset of the disease, to delay its occurrence or possibly halt its progression. To date, only a few common susceptibility alleles for sporadic DCM were identified from candidate-gene approaches, but could not be confirmed in replication samples [2], [3], this being a common problem of single gene based analyses [4]. In contrast, unbiased genome-wide association studies (GWAS) allow the identification of genetic risk factors even outside of known genes, but higher power is needed to compensate for multiple testing [5]. No comprehensive GWAS was performed to date on sporadic form of DCM.

The cardiovascular gene-centric 50K single nucleotide polymorphism (SNP) ITMAT-Broad-CARe (IBC) array represents an established compromise between GWAS and hypothesis-driven candidate gene approach by analyzing polymorphisms in more than 2,000 genes known or predicted to be involved in cardiovascular phenotypes [6].

In this study, we conducted a screening based on the cardiovascular 50K SNP array with three independent replication studies to reveal insight in genetic contribution to idiopathic DCM. The four samples from Germany and France included 1,910 sporadic DCM cases and 3,630 healthy controls individuals. We identified a common intronic variant in *HSPB7*, encoding a cardiovascular small heat shock protein, to be associated with sporadic form of DCM.

## Results

### Screening stage

In our screening case-control sample, DCM cases were more likely men, were slightly younger and less frequently smokers, had a lower BMI and a higher prevalence of hypertension, hypercholesterolemia as well as type 2 diabetes (Table 1).

**Table 1:** Characteristics of DCM cases and controls used for initial screening.

Variable	DCM cases	DCM-free controls	*p*-value
	(*n* = 664)	(*n* = 1,874)	
Gender, % male (*n*)	85.1 (565)	47.4 (888)	<0.0001
Age at diagnosis/inclusion, years	45.6±11.3 (6–70)	62.0±10.9 (35–84)	<0.0001
LVEF, %	24.3±8.7	n. a.	-
Hypertension^a^, % (*n*)	96.2 (639)	57.7 (1,076)	<0.0001
Hypercholesterolemia^b^, % (*n*)	38.1 (253)	29.3 (549)	<0.0001
Type 2 diabetes^c^, % (*n*)	20.6 (137)	9.6 (179)	<0.0001
Smoking^d^, % (*n*)	35.1 (233)	50.8 (952)	<0.0001
BMI, kg/m^2^	26.5±4.6 (15.9–46.7)	28.0±4.5 (16.8–51.4)	<0.0001

Values denote means ± standard deviations (range) unless indicated otherwise. n. a., not available; LVEF, left ventricular ejection fraction; BMI, body mass index.

a Defined as blood pressure ≥140/90 mmHg or ongoing antihypertensive therapy.

b Defined as LDL cholesterol ≥160 mg/dL or intake of lipid lowering medication.

c Defined as self-reported history of diabetes mellitus or intake of antidiabetic medication.

d Former or current smoking habit including current occasionally smokers.

**Figure 1:**
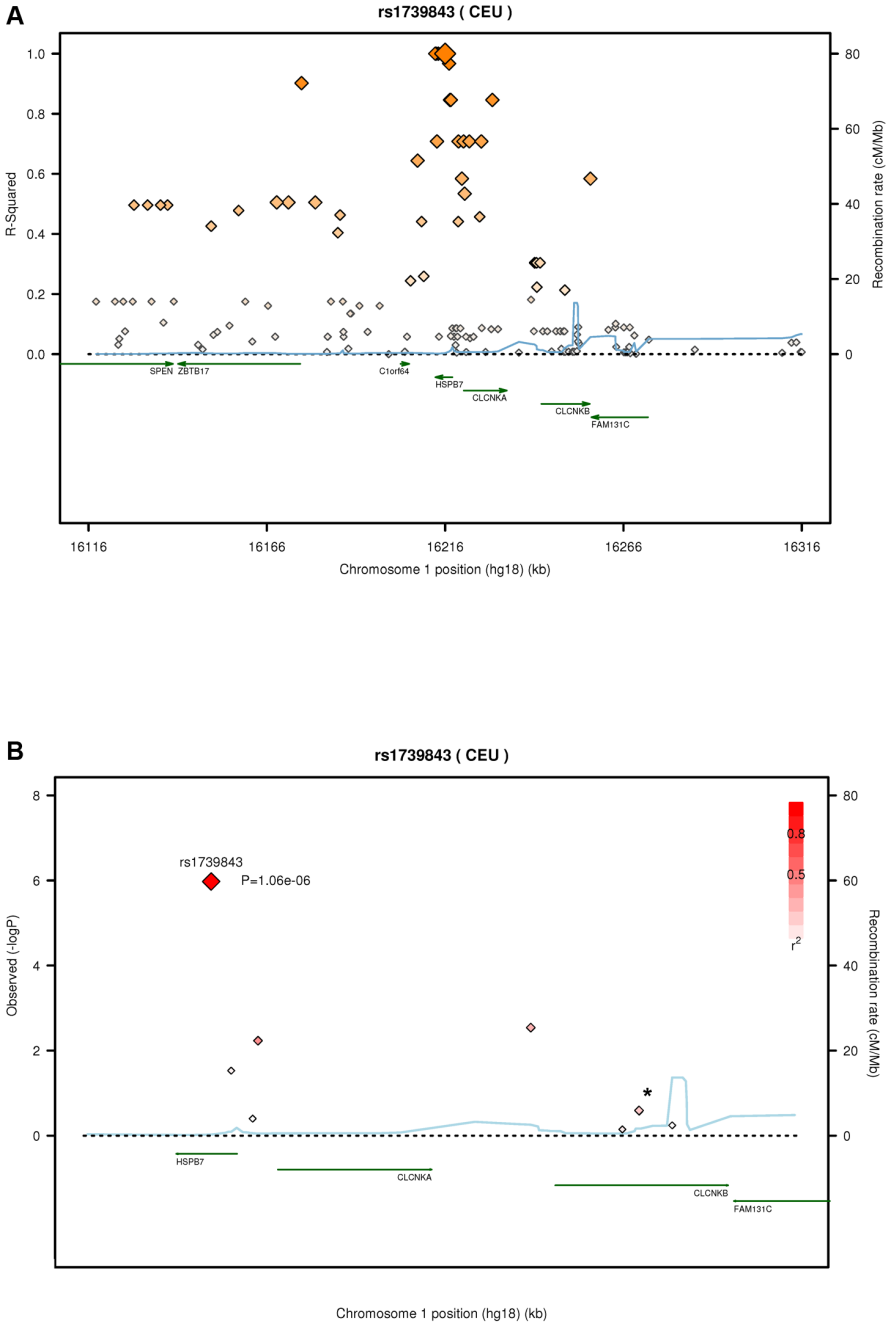
Linkage disequilibrium (LD) structure of *HSPB7* genomic region and association results. (A) LD measurement (r^2^) of HapMap data on CEU samples (release #22) in relation to rs1739843. On each side of the SNP, 100 kb were analyzed and plotted (n = 138 SNPs). (B) SNPs (n = 9, Table S1; *, two SNPs) in *HSPB7* gene region on the 50K gene-centric human CVD bead chip after quality control and λ-corrected association results in 664 DCM cases and 1,874 controls. Plots were generated by using the SNAP tool [33].

After quality control, 30,920 SNPs were available for analysis with 23,307 independent markers (defined as SNPs with pairwise r^2^<0.8 based on linkage disequilibrium (LD) in the control group). Therefore, we set a significance threshold to 0.05/23,307 = 2.15×10^−6^ to account for the multiple testing. In association analyses of this stage 1 study applying logistic regression adjusted for gender, four SNPs, namely rs1739843 (*HSPB7*, intron 2), rs11701453 (*RUNX1*, intron 1), rs7597774 (*ADD2*, intron 1) and rs2229714 (*RPS6KA1*, 3′ untranslated region) showed a p-value below this threshold (3.16*10^−8^, 1.65*10^−7^, 2.05*10^−7^, and 1.51*10^−6^, respectively). Results were similar when additionally adjusting for age (e.g. for rs1739843 p = 2.40*10^−8^). None of the four polymorphisms showed deviation from Hardy-Weinberg equilibrium. The lowest p-value for association with DCM was observed for a SNP located in *HSPB7* intron 2 (rs1739843) leading to a protective effect of the minor allele (OR = 0.67 [95% CI 0.58–0.77]). Analysis of the region around the SNP rs1739843 using HapMap data (release #22) revealed the presence of six genes and 27 polymorphisms in LD with the lead SNP (r^2^-value>0.5) (Figure 1A). Nine of these SNPs were present on the cardiovascular 50K array after quality control and were located in *HSPB7* gene as well as two genes downstream, *CLCNKA* and *CLCNKB* (Figure 1B; Table S1).

In this sample, the genomic inflation factor λ was 1.285 for the highest 90% of the 30,920 observed p-values. When correcting rs1739843 for this λ factor, the p-value was 1.06*10^−6^ and OR = 0.67 [95% CI 0.57–0.79] (Table 2).

**Table 2:** Association of SNPs showing p-values < 2×10^−6^ in the initial screening sample and follow-up in three independent replication samples analyzed by logistic regression adjusted for gender.

			p-value (OR [95% CI]) in screening and replication samples				Combined analysis (n = 5,540)
SNP ID	Nearest gene (RefSeq, SNP location)	Minor allele (frequency)^a^	Screening Germany (n = 664 cases, n = 1,874 controls)^b^	Replication Germany (n = 564 cases, n = 981 controls)	Replication France 1 (n = 433 cases, n = 395 controls)	Replication France 2 (n = 249 cases, n = 380 controls)	p-value (OR [95% CI])^b^
rs1739843	*HSPB7* (NM_014424.4, intron 2)	T (0.39)	1.06*10^−6^ (0.67 [0.57–0.79])	2.07*10^−3^ (0.79 [0.67–0.92])	3.73*10^−3^ (0.74 [0.60–0.91])^c^	2.26*10^−4^ (0.63 [0.50–0.81])	5.28*10^−13^ (0.72 [0.65–0.78])
rs11701453	*RUNX1* (NM_001754.4, intron 1)	C (0.21)	3.88*10^−6^ (1.52 [1.27–1.82])	0.373 (1.09 [0.90–1.31])	0.283 (0.88 [0.69–1.12])	0.723 (0.95 [0.72–1.25])	9.23*10^−3^ (1.15 [1.04–1.28])
rs7597774	*ADD2* (NM_017488.2, intron1)	C (0.36)	4.60*10^−6^ (1.44 [1.23–1.69])	0.621 (1.04 [0.89–1.22])	0.405 (0.92 [0.74–1.13])	0.655 (1.06 [0.83–1.35])	6.16*10^−3^ (1.14 [1.04–1.24])
rs2229714	*RPS6KA1* (NM_002953.3, 3′ UTR)	A (0.15)	2.21*10^−5^ (1.54 [1.26–1.88])	0.075 (1.20 [0.98–1.47])	0.543 (1.10 [0.82–1.47])	0.660 (0.92 [0.64–1.32])	1.98*10^−4^ (1.26 [1.11–1.42])

OR, odds ratio; CI, confidence interval; UTR, untranslated region.

a For screening case-control sample.

b Initial screening sample corrected for λ = 1.285; combined analysis based on the beta-estimates of all four studies using a fixed effect model.

c Proxy SNP rs1763601 was genotyped (HapMap phase 2 release 24: r^2^ between rs1739843 and rs1763601 = 1.0).

### Replication

The four SNPs with uncorrected p<2.15×10^−6^ in the initial scan (rs1739843, rs11701453, rs7597774 and rs2229714) were analyzed using logistic regression adjusted for gender in three independent replication samples. First, n = 564 additional German DCM patients and n = 981 controls were genotyped for the four SNPs. Marker rs1739843 showed strong association with DCM (p = 2.07*10^−3^, OR = 0.79 [95% CI 0.67–0.92]). Conversely, for rs2229714 (p = 0.075, OR = 1.20 [95% CI 0.98–1.47]), rs11701453 (p = 0.373, OR = 1.09 [95% CI 0.90–1.31]) and rs7597774 (p = 0.621, OR = 1.04 [95% CI 0.89–1.22]) the initial association results were not replicated. Second, a French replication sample (France 1) consisted of n = 433 cases and n = 395 controls. Only rs1739843 showed association with DCM after adjustment for gender (p = 3.73*10^−3^, OR = 0.74 [95% CI 0.60–0.91]). For the other SNPs, no significant association was seen in this sample. Third, in an independent second French replication sample (France 2), again only rs1739843 showed association with DCM after adjustment for gender (p = 2.26*10^−4^, OR = 0.63 [95% CI 0.50–0.81]). Replication results are summarized in Table 2. None of the four polymorphisms showed deviation from Hardy-Weinberg equilibrium in any replication samples.

**Figure 2:**
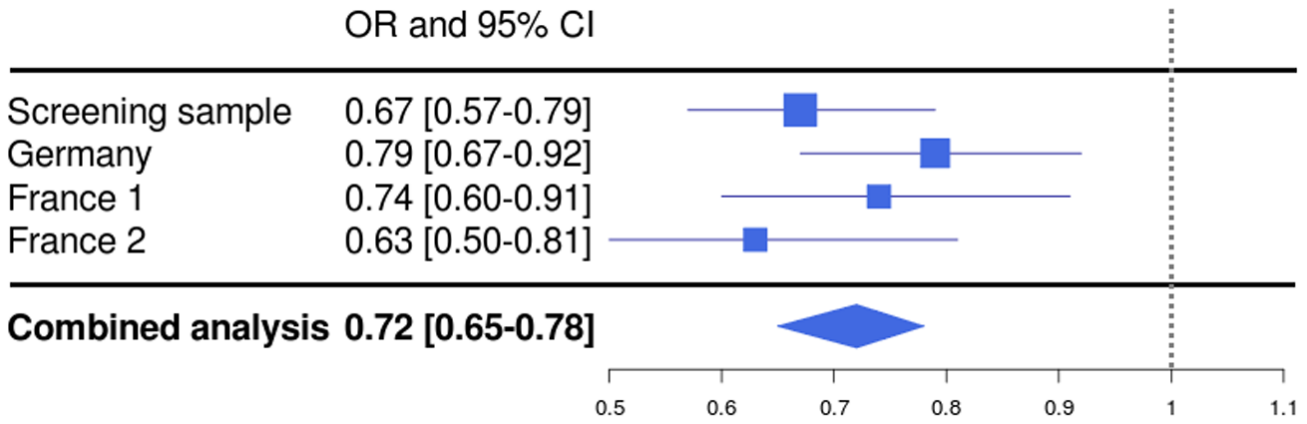
Forest plot for rs1739843 in initial screening sample (λ-corrected) and three replication samples (Germany, France 1, and France 2), together with results from the combined analysis.

In a combined analysis of the screening step, corrected for the λ factor of 1.285, and the three follow-up studies (n = 5,540), the SNP rs1739843 reached a p-value of 5.28*10^−13^ (OR = 0.72 [95% CI 0.65–0.78]) for association with idiopathic DCM (Table 2, Figure 2). There was no between-study heterogeneity for this effect (I^2^ = 6.9%, p = 0.36).

### Resequencing

To reveal potential causal variants, the coding region of *HSPB7* was resequenced in a total of 48 DCM patients. We detected three known synonymous variants (rs945416, rs732286 and rs1739840). The synonymous variants rs945416 (position 19, serine) and rs732286 (position 33, alanine) are in high LD with rs1739843 (r^2^ = 0.96, HapMap CEU data release #24). SNP rs1739840 (position 117, threonine) is not available in HapMap. In the initial sample of 664 DCM patients, all three synonymous polymorphisms are in perfect LD to each other and to rs1739843 as shown by genotyping. Neither missense nor splice site *de novo* mutations were identified by sequencing. Synonymous SNP rs11807575, as well as non-synonymous variants rs77021870 and rs74626772 were listed in databases, but not found to be polymorphic in our sample.

### Analysis of DCM candidate genes

Since the design of the 50K human gene-centric bead chip (IBC array) aims at a large-scale gene-based approach, we screened candidate genes which are known for or potentially involved in susceptibility to DCM in our initial screening sample utilizing the information on 30,920 SNPs. We established a list of previously reported genes for DCM by searching PubMed and OMIM databases (http://www.ncbi.nlm.nih.gov/) for “CARDIOMYOPATHY, DILATED” and “GENETIC”. A total of 315 SNPs including 234 independent SNPs (defined as SNPs with pairwise r^2^<0.8 based on LD in the control group) were located in or near (+/−10kb) the chosen candidate genes, representing 1.01% of array content. DCM association results for these SNPs were obtained from our screening study on 664 cases and 1,874 controls (Table 3, more details in Table S2). On a single candidate gene level, polymorphisms in or near *ABCC9*, *DES*, *MYH6* and *TPM1* showed nominal significance after Bonferroni correction for the number of SNPs tested in gene regions (p = 0.010, p = 0.022, p = 0.005 and p = 0.018, respectively). However, none of these SNPs remained significant after correction for the 234 independent markers tested in this DCM candidate gene approach. Our study was powered to detect moderate to large effects (e. g. for OR>1.3 and MAF = 30% or OR>1.5 and MAF = 20% or OR>1.7 and MAF = 10%, the power was 56%, 96% and 97% for two-sided p<0.05/234 = 2.14*10^−4^, respectively).

**Table 3:** Candidate gene approach on DCM causing or susceptibility genes.

Gene	Protein	Chromosome	Number of SNPs analyzed in gene region +/−10 kb before/after quality control	Best p-value^a^	p_corr_ Bonferroni region-wide after quality control	OMIM	References^b^
*ABCC9 (SUR2A)*	ATP-binding cassette transporter sub-family C member 9	12p12.1	34/25	0.0004	0.010	601439	[20], [34]
*ACTC1*	Actin, alpha, cardiac muscle 1	15q14	13/13	0.1165	1	102540	[20], [35]
*ACTN2*	Actinin, alpha-2	1q43	0	-	-	102573	[20], [36]
*ANKRD1*	Ankyrin repeat domain-containing protein 1, cardiac (CARP)	10q23.31	0	-	-	609599	[37], [38]
*CSRP3*	Cysteine and glycine-rich protein 3 (muscle LIM protein)	11p15.1	13/11	0.0115	0.123	600824	[20], [39]
*DES*	Desmin	2q35	5/3	0.0072	0.022	125660	[20], [40]
*DMD*	Dystrophin	Xp21.2-p21.1	4/4	0.1504	0.602	300377	[20], [41], [42]
*DSG2*	Desmoglein-2	18q12.1	24/16	0.1820	1	125671	[43]
*EYA4*	Eyes absent homolog 4	6q23.2	0	-	-	603550	[44]
*FKTN (FCMD)*	Fukutin	9q31.2	0	-	-	607440	[45]
*HBEGF*	Heparin-binding EGF-like growth factor	5q31.3	11/7	0.1160	0.812	126150	[46]
*IK*	IK cytokine, down-regulator of HLAII (protein RED)	5q31.3	0	-	-	600549	[46]
*LAMA4*	Laminin subunit alpha-4	6q21	0	-	-	600133	[47]
*LDB3*	LIM domain-binding protein 3 (Cypher)	10q23.2	0	-	-	605906	[20]
*LMNA*	Lamin A/C	1q22	7/7	0.3028	1	150330	[20], [48]
*MYBPC3*	Myosin-binding protein C, cardiac	11p11.2	19/9	0.0112	0.101	600958	[20], [49]
*MYH6*	Myosin-6	14q11.2	1/1	0.0049	0.005	160710	[20], [50]
*MYH7*	Myosin-7	14q11.2	3/3	0.2691	0.807	160760	[20], [51]
*NEBL*	Nebulette (Actin-binding Z-disk protein)	10p12.31	2/2	0.3159	0.632	605491	[52]
*NEXN*	Nexilin (F actin binding protein)	1p31.1	6/5	0.4556	1	613121	[53]
*PLN*	Phospholamban	6q22.31	4/3	0.0465	0.140	172405	[20], [54], [55]
*PSEN1*	Presenilin-1	14q24.2	0	-	-	104311	[56]
*PSEN2*	Presenilin-2	1q42.13	8/6	0.2753	1	600759	[56]
*RBM20*	Ribonucleic acid binding motif protein 20	10q25.2	0	-	-	613171	[57]
*SCN5A*	Sodium channel protein type 5 subunit alpha	3p22.2	51/42	0.0291	1	600163	[58]
*SGCD*	Sarcoglycan delta	5q33.3	84/64	0.0182	1	601411	[20], [59]
*SRA1*	Steroid receptor RNA activator 1	5q31.3	0	-	-	603819	[46]
*TAZ*	Tafazzin	Xq28	4/4	0.1633	0.653	300394	[20], [60], [61]
*TCAP*	Telethonin	17q12	8/5	0.4175	1	604488	[2], [62]
*TMPO*	Thymopoietin	12q23.1	12/8	0.0260	0.208	188380	[63]
*TNNC1*	Troponin C, slow skeletal and cardiac muscles	3p21.1	4/4	0.0794	0.318	191040	[64]
*TNNI3*	Troponin I, cardiac muscle	19q13.42	14/10	0.0266	0.266	191044	[20], [65]
*TNNT2*	Troponin T, cardiac muscle	1q32.1	15/12	0.0081	0.097	191045	[20], [51]
*TPM1*	Tropomyosin alpha-1	15q22.2	11/10	0.0018	0.018	191010	[20], [66]
*TTN*	Titin	2q31.2	43/28	0.0404	1	188840	[20], [67]
*VCL*	Vinculin	10q22.2	19/13	0.1731	1	193065	[20], [68]

a Logistic regression adjusting for gender.

b Review, original publication(s).

Details are listed in Table S2.

## Discussion

In the present case-control study, we evaluated the relationship of common SNPs with sporadic DCM using a large-scale screening approach. Our comprehensive strategy set out to analyze the human gene-centric 50K bead chip (IBC array), which focuses on loci with a potential functional link to cardiovascular disease (CVD) and covers more than 45,000 SNPs from about 2,000 genes [6].

Our study identified a polymorphism (rs1739843) in intron 2 of the *HSPB7* gene being associated with susceptibility to DCM in a German case-control sample with three replication steps. Recently, Cappola et al. reported an association between rs1739843 and both, ischemic and non-ischemic heart failure, applying the same gene-centric 50K bead chip [7]. They found a protective effect of the minor allele, which is in conformity with our results on DCM. As DCM is a potential preliminary stage for non-ischemic heart failure, these independent findings point to a possible common pathophysiologic cascade. However, a second association signal for heart failure located in the *FRMD4B* region (rs6787362, minor allele frequency (MAF) 10.4%) identified by Cappola et al. [7] could not be detected in our DCM case-control sample (p = 0.64). Our study had a power of 99% to find a nominal association between DCM and rs6787362 with p<0.05 and an OR = 0.67.

The finding on *HSPB7* is also in-line with a previously reported large-scale re-sequencing approach in four biologically relevant cardiac signaling genes, which detected *HSPB7* sequence diversity in sporadic cardiomyopathy [8]. Our data together with the results from Cappola et al. [7] and Matkovich et al. [8], substantiate the importance of rs1739843 or related polymorphisms in the *HSPB7* locus for DCM and heart failure and possibly underscore a common genetic basis for these related phenotypes.

Matkovich et al. further report that none of the detected *HSPB7* gene variants altered amino acid sequence [8], which is also consistent with the fact that we found neither missense nor splice site mutations in the *HSPB7* sequence. Therefore, the biological mechanism explaining the association between the polymorphism rs1739843 and DCM risk remains still unclear. The three detected synonymous variants (rs945416, rs732286 and rs1739840) are in high LD with each other as well as with our lead SNP rs1739843 and lie on one LD block. Therefore, it could be hypothesized that these SNPs represent causal risk factors for DCM, as described for the P-glycoprotein encoding gene *MCP1* and affected drug and inhibitor interactions [9]. Synonymous SNPs lead to changes in codon usage and may cause functional implications by conformational changes in protein structure due to translation efficiency. Alternatively, a *de novo* splice site could be created by a SNP or other (unmapped) polymorphisms outside the *HSPB7* coding region may alter its gene expression. Clearly, functional studies would be required to prove these hypotheses.

Besides the *HSPB7* gene, where the lead SNP is located, also five genes (*CLCNKA*, *CLCNKB*, *C1orf64*, *ZBTB17* and *SPEN*) lying on the same LD block may potentially be responsible for the association with DCM. *CLCNKA* and *CLCNKB* encode for two members of the family of voltage-gated chloride channels. These proteins are predominantly expressed in the kidney and participate in renal salt reabsorption [10]. The function of *C1orf64* is currently unknown. *ZBTB17*, also known as *MIZ-1*, encodes a zinc finger protein involved in the regulation of c-myc [11]. *SPEN* (*RBM15C* or *MINT*) encodes a conserved transcriptional repressor that controls the expression of regulators in diverse signaling pathways [12], [13].

*HSPB7*, encoding the small heat shock protein cvHsp (also known as HspB7), is the functionally most plausible candidate gene in this genomic region. It is known to be expressed in cardiovascular and insulin-sensitive tissues [14]. In general, the expression and activation of heat shock proteins is influenced by elevated temperatures as well as ischemia, hypoxia and acute cellular stress [15], [16]. In the aging skeletal muscle increase of cvHsp protein content was observed [17]. cvHsp was shown to be constitutively localized under non-stressful conditions to nuclear splicing speckles and may influence mRNA processing [18]. Recent data suggest co-localization between cvHsp and α-B-crystallin in the z-band of cardiac tissue and interaction with other small heat shock proteins [19]. However, further investigations like genomic fine-mapping and subgroup analyses in the context of cardiomyopathies are needed.

Genetic analyses in familial forms of DCM led to the identification of risk loci showing X-linked, autosomal dominant or autosomal recessive patterns of inheritance [2], [20], [21]. Some of the DCM causing genes or plausible candidate genes were also covered by the 50K bead chip, wherefore we specifically tested those SNPs lying in risk gene regions (10 kb upstream and downstream, respectively). In these analyses, no significant association with any of the gene variants was found, indicating that in sporadic cases of DCM probably other pathways are involved than in familial DCM. However, less frequent variants may have been missed due to insufficient power of our screening sample. Furthermore, the distinction between familial and sporadic forms of DCM is, to a certain degree, somewhat arbitrary. Screening of family members is rarely done in clinical routine, but when carried out on a systematic basis, up to 7% of previously healthy first-degree relatives have reduced left ventricular function or dilation without presence of cardiac symptoms [22]. Therefore, it might be anticipated that genetic testing could help to identify individuals at risk in familial DCM but also in families of patients affected by so-called idiopathic forms of the disease.

Already known genetic factors account for only a fraction of DCM heritability [20]. Given a 1.5-fold increased risk of DCM among heterozygous subjects in our screening sample (48% in the general population-based KORA study) and a 2.25 times increased risk among homozygous subjects (34% in KORA), 49% of DCM cases would be attributable to the SNP rs1739843 (or correlated polymorphisms) with 19% attributable to heterozygous and 30% to homozygous carriers, respectively. Therefore, the genetic component seems to comprise a large proportion for this disease. However, with the prevalence of the idiopathic form of the disease being about 1∶2,700 [23], a genetic screening of the general population would include four cases out of 10,000 screened persons and two of these would have the disease due to this SNP. Therefore, the great potential of this variant might rather be screening of high risk populations, or this pathway indicates potential drug targets. Further investigations should aim (1) to identify additional variants underlying DCM susceptibility with otherwise unknown etiology and (2) to analyze potential influence of these common alleles as modifiers for familial forms of DCM. Taken together for both, modifiers of familial forms and susceptibility alleles in idiopathic DCM, knowledge of genetic background will support preventive medical measures in the future.

Some limitations of our study should be mentioned. First, we conducted a large-scale SNP analysis focused on genes potentially involved in cardiovascular traits. Therefore, on the one hand we were able to detect associations between DCM and polymorphisms only in these pre-selected genes. On the other hand, the 50K human CVD bead chip allows comprehensive gene-based analysis with more than 2,000 well covered loci. Second, our sample size only allowed to detect moderate to large effects (e. g. for OR>1.3 and MAF = 30% or OR>1.5 and MAF = 20% or OR>1.7 and MAF = 10%, the power was 19%, 75% and 80% for p<2.15*10^−6^, respectively). Therefore, we may have overlooked real association signals in our screening step. Third, there could be some population stratification in our initial screen sample. However, the observed λ could also be caused - in part - by underlying association due to the analysis of pre-selected loci known or suggested to be involved in cardiovascular phenotypes. The fact that the association between rs1739843 in *HSPB7* and idiopathic DCM was replicated in three independent samples strongly enhances the confidence in our results.

## Materials and Methods

### Ethics statement

The ethics committees of the participating study centers approved the study protocol and all participants gave their written informed consent. The study was in accordance with the principles of the current version of the Declaration of Helsinki.

### Case-control samples and phenotyping

Cases for the initial German screening study were recruited from the German Heart Institute (Berlin), and controls were from a population-based German KORA study (follow-up survey F3, Augsburg) [24]. Phenotypic details are summarized in Table 1. Controls (n = 1,874) had no medical history for coronary artery disease (CAD), myocardial infarction or DCM; mean age was 62±11 years and slightly more women (n = 986) than men (n = 888) were present in the control group. Inclusion criteria for DCM cases were the following: reduced systolic function (left ventricular ejection fraction (LVEF) <45%) without angiographically assessed evidence of major CAD, significant valvular heart disease (>grade 2, i. e. such as mitral or aortic regurgitation), hypertensive heart disease, congenital heart disease, myocarditis (by endomyocardial biopsy, when available) or other secondary forms of heart failure. Patients with a positive family history were also excluded from this study. In DCM cases (n = 664), mean LVEF was 24±3% and mean age of disease diagnosis was 46±11 years.

For the first replication step, additional German DCM cases (mean age 53±13 years; n = 564, n = 440 men, n = 124 women) were recruited from different German study centers: Berlin, n = 64; Lübeck, n = 96 (Angio-Lueb); Marburg, n = 61 (EUROGENE); Münster, n = 101 (EUROGENE); Regensburg, n = 150 (EUROGENE); Regensburg, n = 92 (GoKard). Independent German KORA controls from surveys S1 and S2 (n = 981, n = 539 men, n = 442 women) had a mean age of 52±10 years [24]. Inclusion and exclusion criteria were identical to the initial case-control sample.

A second replication study (France 1) was recruited in France (CARDIGENE) [25], [26]. The French cases were of white European origin (all born in France, from parents born in France or neighboring countries) with a diagnosis of DCM, i. e. enlarged left ventricle end-diastolic volume/diameter >140 ml/m^2^ on ventriculography or >34 mm/m^2^ on echocardiography and LVEF ≤40% confirmed over a six-month period, in the absence of causal factors such as CAD or sustained hypertension, intrinsic valvular disease, documented myocarditis, congenital malformation, insulin-dependent diabetes. Only apparently sporadic DCM cases without additional (first degree) relative with DCM were included (but 8% were in fact with familial form after careful cardiac examination in relatives). Recruitment was performed in ten hospitals in six regions in France (Lille, Lyon, Nancy, Nantes, Paris-Ile de France, Strasbourg) from September 1994 to February 1996. A total of 433 patients (229 had undergone a cardiac transplantation) were included (n = 345 men, n = 88 women). Mean age of patients was 45±11 years, mean LVEF was 23±7% and mean end-diastolic volume was 195±67 ml/m^2^. Controls (n = 395) were age- and gender-matched (n = 310 men, n = 85 women).

The third replication sample was also of French origin (France 2). Inclusion criteria were identical to the France 1 sample. A total of 249 patients from EUROGENE and PHRC were included (n = 198 men, n = 51 women). Mean age of patients at diagnosis was 51±10 years. Controls (n = 380) were free of medical history for CAD, myocardial infarction or DCM and mean age was 46±11 years (n = 301 men, n = 79 women).

### Genotyping

Initial genotyping was carried out using the 50K gene-centric human CVD bead chip version 1 (IBC v1 array) (Illumina, San Diego, CA, USA) [6] following the manufacturer's protocol. Data were analyzed (calling and sample clustering) and exported employing BeadStudio analysis software (Illumina). From the initial 45,707 SNPs, those markers with low call rates (<95%) or low frequency (MAF<1%) were excluded. Minimal call rate per individual was 90%. We used identity-by-descent methods to exclude unknown first-degree relation of participants.

Replication samples were taken from human CVD bead chip data or genotyped with 5′ exonuclease TaqMan technology (Applied Biosystems, Foster City, CA, USA) as previously described [27]. A by-design assay for rs1739843 was used with primer sequences 5′-CTCTGCCATCACCATCTCACA-3′ and 5′-GGCAGAGGGAGCCTGAG-3′ and probe sequences 5′-VIC-AGGGTGGGAGGTGACAG-NFQ-3′ and 5′-FAM-AGGGTGGGAGATGACAG-NFQ-3′ (site of rs1739843 is underlined; fluorescence dyes VIC and FAM on 5′ end and non-fluorescence quencher (NFQ) on 3′ end are indicated). All other assays were obtained pre-designed directly from Applied Biosystems. Detailed information on assays used in France 2 sample are available at http://genecanvas.ecgene.net/infusions/genecanvas/Polymorphisms/PolymorphismsList.php.

SNP rs1739843 was re-genotyped using the by-design TaqMan assay in initial case sample (n = 664) to check for discrepancies between human CVD bead chip and TaqMan genotypes. A >99.8% concordance of genotypes was found. For all genotyped samples a call rate >97% for each SNP assay was reached.

### Resequencing

Polymerase chain reaction (PCR) primer were generated using Primer3Plus (http://www.bioinformatics.nl/cgi-bin/primer3plus/primer3plus.cgi) [28] to cover the coding parts of the three *HSPB7* exons (GenBank accession No. NM_14424.4). The primer sequences and PCR amplification products are listed in Table S3. Included intronic regions were 267 bp for 5′ end of intron 1, 156 bp for 3′ end of intron 1, 136 bp for 5′ end of intron 2, and 89 bp for 3′ end of intron 2, respectively. PCR cycling conditions consisted of an initial denaturation at 95°C for 9 min, followed by 40 cycles with denaturation at 95°C for 30 s, annealing at 60°C for 30 s, and elongation at 72°C for 30 s, with a final elongation step at 72°C for 7 min.

After PCR amplification, primers and dNTPs were removed using ExoSAP-IT (USB Europe, Staufen, Germany) following the manufacturer's instructions. The purified PCR products were directly sequenced using the ABI PRISM BigDye Terminator Cycle Sequencing Ready Reaction Kit Version 3.1 on the ABI 3730 (Applied Biosystems, Foster City, CA, USA).

### Statistical analyses

For initial screening and replication analyses, logistic regression adjusted for gender was used. P-values, odds ratios (OR) and their 95% confidence intervals (CI) were reported. The inflation factor λ was computed in the 50K initial screening analysis for logistic regression analysis assuming a χ^2^ distribution with two degrees of freedom of the minus two-times log_e_p measures (90% highest p-values). The p-values and CI from initial screening analysis were genomic-control corrected using this λ factor via standard errors (standard error_[corrected]_ = sqrt(λ)*standard error) and beta estimates (95%CI beta_[corrected]_ = beta±1.96*standard error_[corrected]_). Deviation from Hardy-Weinberg equilibrium was calculated with an exact test [29]. Statistical and association analyses were performed using JMP 7.0.2 (SAS Institute Inc, Cary, NC, USA) and PLINK v1.07 (http://pngu.mgh.harvard.edu/~purcell/plink/) [30], respectively. Power analysis was carried out using Quanto 1.2.4 (http://hydra.usc.edu/gxe/). We combined the initial scan results corrected for λ with the replication studies' results using a fixed effect model. Annotation of association results on a genome level was performed with WGAViewer software (http://people.genome.duke.edu/~dg48/WGAViewer/) [31]. LD patterns were calculated using HapMap releases #22 and #24 (http://www.hapmap.org/) [32].

## Supporting Information

Table S1 Association results of SNPs in *HSPB7* genomic region in initial screening sample (664 cases, 1,874 controls).
(0.05 MB DOC)

Table S2 Association of previously reported DCM causing or susceptibility genes in initial screening sample (664 cases, 1,874 controls).
(0.49 MB DOC)

Table S3 Primer sequences for PCR amplification of the *HSPB7* coding region and product sizes.
(0.03 MB DOC)
