# Supplementary material for: Genetic Association Study Identifies HSPB7 as a Risk Gene for Idiopathic Dilated Cardiomyopathy
Source: PLoS Genet. 2010 Oct 21;6(10):e1001167. doi: 10.1371/journal.pgen.1001167 (PMC2958814; doi:10.1371/journal.pgen.1001167)
Supplement: Table S1 — Association results of SNPs in HSPB7 genomic region in initial screening sample (664 cases, 1,874 controls). (0.05 MB DOC) [file pgen.1001167.s001.doc]

**Table S1.** Association results of SNPs in *HSPB7* genomic region in initial screening sample (664 cases, 1,874 controls)

| Position | SNP | Minor allele | Major allele | MAF | p-value ^a^ | OR (95% CI ^a^) | LD (r²) to rs1739843 |
| --- | --- | --- | --- | --- | --- | --- | --- |
| 16215841 | rs1739843 | T | C | 0.393 | 1.06*10^-6^ | 0.67 (0.57-0.79) | 1 |
| 16217414 | rs1056207 | T | C | 0.104 | 0.030 | 1.31 (1.03-1.67) | 0.081 |
| 16219094 | rs3754322 | G | T | 0.214 | 0.398 | 1.08 (0.90-1.30) | 0 |
| 16219511 | rs2009594 | A | G | 0.408 | 5.85*10^-3^ | 1.24 (1.06-1.45) | 0.444 |
| 16240911 | rs10803410 | A | G | 0.482 | 2.88*10^-3^ | 0.79 (0.68-0.92) | 0.336 |
| 16248097 | rs11588392 | A | G | 0.027 | 0.712 | 1.09 (0.69-1.74) | 0.034 |
| 16249403 | rs10927894 | G | C | 0.258 | 0.255 | 0.90 (0.74-1.08) | 0.209 |
| 16249418 | rs10803412 | C | T | 0.175 | 0.273 | 0.89 (0.73-1.09) | 0 |
| 16252003 | rs12015135 | C | A | 0.115 | 0.571 | 1.07 (0.85-1.35) | 0.015 |

^a^ Corrected for λ=1.285
