# Supplementary material for: Genetic Association Study Identifies HSPB7 as a Risk Gene for Idiopathic Dilated Cardiomyopathy
Source: PLoS Genet. 2010 Oct 21;6(10):e1001167. doi: 10.1371/journal.pgen.1001167 (PMC2958814; doi:10.1371/journal.pgen.1001167)
Supplement: Table S2 — Association of previously reported DCM causing or susceptibility genes in initial screening sample (664 cases, 1,874 controls). (0.49 MB DOC) [file pgen.1001167.s002.doc]

**Table S2.** Association of previously reported DCM causing or susceptibility genes in initial screening sample (664 cases, 1,874 controls)

| **Gene** | **SNP** | **Chromosome** | **Location** | **Rank *** | **p-value** |
| --- | --- | --- | --- | --- | --- |
| *ABCC9* | rs11046238 | 12 | 22092921 | 116 | 0.0004 |
|  | rs1283816 | 12 | 21984711 | 428 | 0.0025 |
|  | rs7978092 | 12 | 22047455 | 760 | 0.0061 |
|  | rs704179 | 12 | 21993620 | 1839 | 0.0230 |
|  | rs1517284 | 12 | 21988088 | 2461 | 0.0346 |
|  | rs1643235 | 12 | 21995555 | 3680 | 0.0609 |
|  | rs12369421 | 12 | 21992297 | 3935 | 0.0665 |
|  | rs3782667 | 12 | 21985006 | 4000 | 0.0678 |
|  | rs704206 | 12 | 22038479 | 4083 | 0.0696 |
|  | rs1352909 | 12 | 22040041 | 4212 | 0.0729 |
|  | rs704217 | 12 | 22047174 | 5086 | 0.0937 |
|  | rs4148674 | 12 | 22003544 | 6212 | 0.1209 |
|  | rs704190 | 12 | 22014473 | 6896 | 0.1398 |
|  | rs4148649 | 12 | 22070231 | 8664 | 0.1917 |
|  | rs1283811 | 12 | 21981225 | 9562 | 0.2199 |
|  | rs4148663 | 12 | 22035615 | 9865 | 0.2288 |
|  | rs2638441 | 12 | 21970019 | 10060 | 0.2346 |
|  | rs11046205 | 12 | 21992326 | 13411 | 0.3441 |
|  | rs1356368 | 12 | 22035873 | 13793 | 0.3578 |
|  | rs10770867 | 12 | 22071795 | 15790 | 0.4272 |
|  | rs12230539 | 12 | 22013213 | 18460 | 0.5206 |
|  | rs16924332 | 12 | 21974228 | 19425 | 0.5562 |
|  | rs7316271 | 12 | 22090462 | 25206 | 0.7751 |
|  | rs1283809 | 12 | 21980618 | 28133 | 0.8893 |
|  | rs1283798 | 12 | 22021195 | 30569 | 0.9816 |
|  |  |  |  |  |  |
| *ACTC1* | rs525720 | 15 | 35089134 | 6027 | 0.1165 |
|  | rs533021 | 15 | 35080931 | 9059 | 0.2038 |
|  | rs475786 | 15 | 35091138 | 9844 | 0.2284 |
|  | rs893132 | 15 | 35090060 | 11875 | 0.2907 |
|  | rs893131 | 15 | 35090912 | 14169 | 0.3720 |
|  | rs7168956 | 15 | 35092378 | 15879 | 0.4300 |
|  | rs2070664 | 15 | 35085201 | 16111 | 0.4382 |
|  | rs7165006 | 15 | 35085897 | 18436 | 0.5196 |
|  | rs893130 | 15 | 35091453 | 22174 | 0.6579 |
|  | rs641563 | 15 | 35091385 | 22294 | 0.6622 |
|  | rs1370154 | 15 | 35082225 | 25594 | 0.7911 |
|  | rs746216 | 15 | 35088292 | 25620 | 0.7920 |
|  | rs670957 | 15 | 35089432 | 26891 | 0.8440 |
|  |  |  |  |  |  |
| *CSRP3* | rs1077688 | 11 | 19208070 | 1144 | 0.0115 |
|  | rs10766533 | 11 | 19224677 | 2048 | 0.0265 |
|  | rs721067 | 11 | 19212726 | 2305 | 0.0314 |
|  | rs1346117 | 11 | 19223051 | 6589 | 0.1298 |
|  | rs13451 | 11 | 19207841 | 9986 | 0.2328 |
|  | rs1346118 | 11 | 19219499 | 12128 | 0.2993 |
|  | rs10741762 | 11 | 19223143 | 12963 | 0.3291 |
|  | rs10766530 | 11 | 19210112 | 14470 | 0.3821 |
|  | rs6483581 | 11 | 19209135 | 18433 | 0.5194 |
|  | rs10741763 | 11 | 19224718 | 18991 | 0.5392 |
|  | rs3781799 | 11 | 19208319 | 28354 | 0.8973 |
|  |  |  |  |  |  |
| *DES* | rs1058284 | 2 | 220286142 | 838 | 0.0072 |
|  | rs6436151 | 2 | 220297176 | 29101 | 0.9257 |
|  | rs907684 | 2 | 220299272 | 30256 | 0.9697 |
|  |  |  |  |  |  |
| *DMD* | rs5927163 | X | 33357592 | 7271 | 0.1504 |
|  | rs10522005 | X | 32561468 | 9148 | 0.2063 |
|  | rs1293916 | X | 32023059 | 17446 | 0.4844 |
|  | rs6631456 | X | 32028657 | 26096 | 0.8111 |
|  |  |  |  |  |  |
| *DSG2* | rs2256638 | 18 | 29101803 | 8338 | 0.1820 |
|  | rs2230234 | 18 | 29104714 | 8899 | 0.1988 |
|  | rs3737375 | 18 | 29104204 | 11794 | 0.2877 |
|  | rs2187047 | 18 | 29098635 | 12081 | 0.2975 |
|  | rs1791173 | 18 | 29119357 | 13355 | 0.3419 |
|  | rs10163532 | 18 | 29111449 | 14614 | 0.3864 |
|  | rs2230233 | 18 | 29104698 | 15712 | 0.4247 |
|  | rs9962121 | 18 | 29076717 | 20298 | 0.5892 |
|  | rs9304098 | 18 | 29083630 | 21097 | 0.6179 |
|  | rs2212618 | 18 | 29088148 | 22609 | 0.6748 |
|  | rs2848675 | 18 | 29089885 | 25421 | 0.7837 |
|  | rs1042769 | 18 | 29125854 | 25462 | 0.7858 |
|  | rs8084771 | 18 | 29101686 | 28367 | 0.8977 |
|  | rs9947401 | 18 | 29105117 | 29561 | 0.9422 |
|  | rs1941939 | 18 | 29109642 | 30660 | 0.9856 |
|  | rs12326068 | 18 | 29111364 | 30872 | 0.9940 |
|  |  |  |  |  |  |
| *HBEGF* | rs2074613 | 5 | 139714564 | 5994 | 0.1160 |
|  | rs7268 | 5 | 139712550 | 7637 | 0.1604 |
|  | rs4150196 | 5 | 139726641 | 10403 | 0.2456 |
|  | rs13385 | 5 | 139712878 | 12346 | 0.3070 |
|  | rs3776089 | 5 | 139727260 | 13112 | 0.3343 |
|  | rs4912711 | 5 | 139718525 | 23041 | 0.6908 |
|  | rs1862176 | 5 | 139730444 | 29695 | 0.9476 |
|  |  |  |  |  |  |
| *LMNA* | rs584025 | 1 | 156088526 | 12237 | 0.3028 |
|  | rs501791 | 1 | 156089873 | 12589 | 0.3158 |
|  | rs577492 | 1 | 156100739 | 14718 | 0.3904 |
|  | rs2485662 | 1 | 156083468 | 17629 | 0.4909 |
|  | rs476000 | 1 | 156106369 | 19419 | 0.5562 |
|  | rs6686943 | 1 | 156092991 | 22305 | 0.6625 |
|  | rs9427236 | 1 | 156093406 | 28479 | 0.9023 |
|  |  |  |  |  |  |
| *MYBPC3* | rs3729989 | 11 | 47370041 | 1120 | 0.0112 |
|  | rs11570067 | 11 | 47366095 | 1981 | 0.0253 |
|  | rs2290149 | 11 | 47345916 | 4614 | 0.0823 |
|  | rs3729986 | 11 | 47371598 | 7830 | 0.1661 |
|  | rs11570094 | 11 | 47359706 | 8055 | 0.1735 |
|  | rs2697920 | 11 | 47370607 | 11219 | 0.2704 |
|  | rs11570115 | 11 | 47354905 | 21679 | 0.6399 |
|  | rs753992 | 11 | 47349846 | 23135 | 0.6944 |
|  | rs11039185 | 11 | 47349699 | 26002 | 0.8076 |
|  |  |  |  |  |  |
| *MYH6* | rs365990 | 14 | 23861811 | 667 | 0.0049 |
|  |  |  |  |  |  |
| *MYH7* | rs12590407 | 14 | 24835115 | 11167 | 0.2691 |
|  | rs10148620 | 14 | 24830749 | 27587 | 0.8698 |
|  | rs2229309 | 14 | 24839083 | 29373 | 0.9357 |
|  |  |  |  |  |  |
| *NEBL* | rs7079790 | 10 | 21296963 | 12591 | 0.3159 |
|  | rs604251 | 10 | 21323423 | 14360 | 0.3788 |
|  |  |  |  |  |  |
| *NEXN* | rs17101082 | 1 | 78401207 | 16627 | 0.4556 |
|  | rs1166698 | 1 | 78392446 | 23281 | 0.6997 |
|  | rs1780047 | 1 | 78403856 | 23654 | 0.7146 |
|  | rs1166702 | 1 | 78395562 | 30605 | 0.9834 |
|  | rs1780050 | 1 | 78400540 | 30769 | 0.9900 |
|  |  |  |  |  |  |
| *PLN* | rs3752581 | 6 | 118869730 | 3040 | 0.0465 |
|  | rs9489438 | 6 | 118877470 | 6759 | 0.1354 |
|  | rs9481825 | 6 | 118876092 | 12905 | 0.3271 |
|  |  |  |  |  |  |
| *PSEN2* | rs1800680 | 1 | 227075984 | 11383 | 0.2753 |
|  | rs2073489 | 1 | 227063671 | 17210 | 0.4764 |
|  | rs1800681 | 1 | 227076223 | 19296 | 0.5508 |
|  | rs1295645 | 1 | 227059037 | 20266 | 0.5874 |
|  | rs12070751 | 1 | 227082438 | 26014 | 0.8081 |
|  | rs1046240 | 1 | 227071525 | 26344 | 0.8220 |
|  |  |  |  |  |  |
| *SCN5A* | rs12498069 | 3 | 38660936 | 2178 | 0.0291 |
|  | rs6768664 | 3 | 38684466 | 4923 | 0.0901 |
|  | rs7373102 | 3 | 38680628 | 5653 | 0.1071 |
|  | rs9311190 | 3 | 38627930 | 6766 | 0.1356 |
|  | rs11710077 | 3 | 38657899 | 7115 | 0.1462 |
|  | rs7624535 | 3 | 38665202 | 7945 | 0.1700 |
|  | rs1805126 | 3 | 38592406 | 8251 | 0.1797 |
|  | rs3934936 | 3 | 38664309 | 9274 | 0.2102 |
|  | rs7427106 | 3 | 38678501 | 9412 | 0.2147 |
|  | rs7633974 | 3 | 38665923 | 9665 | 0.2229 |
|  | rs6763048 | 3 | 38681394 | 10303 | 0.2429 |
|  | rs12491987 | 3 | 38649044 | 10669 | 0.2542 |
|  | rs1805124 | 3 | 38645420 | 11098 | 0.2672 |
|  | rs12053903 | 3 | 38593393 | 11217 | 0.2704 |
|  | rs9311195 | 3 | 38681218 | 11912 | 0.2921 |
|  | rs10154914 | 3 | 38632630 | 12711 | 0.3206 |
|  | rs6599229 | 3 | 38672585 | 13549 | 0.3494 |
|  | rs6770569 | 3 | 38647780 | 14762 | 0.3919 |
|  | rs13084981 | 3 | 38645999 | 15306 | 0.4114 |
|  | rs7427874 | 3 | 38643279 | 16092 | 0.4376 |
|  | rs7373819 | 3 | 38619312 | 16300 | 0.4444 |
|  | rs9832586 | 3 | 38607078 | 20881 | 0.6103 |
|  | rs9876660 | 3 | 38664715 | 21024 | 0.6153 |
|  | rs6599225 | 3 | 38653277 | 21276 | 0.6253 |
|  | rs6781731 | 3 | 38663707 | 23257 | 0.6988 |
|  | rs3935472 | 3 | 38602855 | 23502 | 0.7088 |
|  | rs9861242 | 3 | 38609334 | 23524 | 0.7096 |
|  | rs6797133 | 3 | 38656033 | 24280 | 0.7386 |
|  | rs9832895 | 3 | 38661533 | 24417 | 0.7440 |
|  | rs9833086 | 3 | 38610471 | 24469 | 0.7465 |
|  | rs9831389 | 3 | 38652884 | 25677 | 0.7950 |
|  | rs9809363 | 3 | 38689231 | 26115 | 0.8119 |
|  | rs6768135 | 3 | 38678009 | 26530 | 0.8300 |
|  | rs7374605 | 3 | 38607303 | 26811 | 0.8407 |
|  | rs9812912 | 3 | 38607229 | 27195 | 0.8557 |
|  | rs7373934 | 3 | 38695708 | 27429 | 0.8642 |
|  | rs7372712 | 3 | 38686192 | 27655 | 0.8726 |
|  | rs7374138 | 3 | 38605732 | 28104 | 0.8883 |
|  | rs7433206 | 3 | 38657708 | 28949 | 0.9198 |
|  | rs7427447 | 3 | 38606588 | 30069 | 0.9622 |
|  | rs7374540 | 3 | 38634142 | 30352 | 0.9736 |
|  | rs13073578 | 3 | 38679364 | 30623 | 0.9840 |
|  |  |  |  |  |  |
| *SGCD* | rs1504933 | 5 | 155870422 | 1555 | 0.0182 |
|  | rs10038955 | 5 | 155748904 | 2991 | 0.0456 |
|  | rs983467 | 5 | 155886268 | 3300 | 0.0528 |
|  | rs6556615 | 5 | 155859368 | 3934 | 0.0665 |
|  | rs6877118 | 5 | 156039988 | 4248 | 0.0736 |
|  | rs256826 | 5 | 155935991 | 4347 | 0.0761 |
|  | rs157664 | 5 | 156123995 | 5040 | 0.0925 |
|  | rs924872 | 5 | 155813324 | 5097 | 0.0941 |
|  | rs157670 | 5 | 156113221 | 5595 | 0.1058 |
|  | rs2055611 | 5 | 155863703 | 6095 | 0.1182 |
|  | rs17559950 | 5 | 155997518 | 6477 | 0.1274 |
|  | rs10071452 | 5 | 155971647 | 7368 | 0.1528 |
|  | rs4704804 | 5 | 155907487 | 7624 | 0.1600 |
|  | rs6883496 | 5 | 155941078 | 7889 | 0.1677 |
|  | rs10069144 | 5 | 155999157 | 8480 | 0.1859 |
|  | rs891906 | 5 | 155940126 | 8614 | 0.1903 |
|  | rs284445 | 5 | 156187928 | 9327 | 0.2117 |
|  | rs11740347 | 5 | 155756414 | 9405 | 0.2144 |
|  | rs466781 | 5 | 156154144 | 9424 | 0.2151 |
|  | rs282474 | 5 | 156141917 | 10770 | 0.2568 |
|  | rs17053865 | 5 | 156154478 | 11085 | 0.2668 |
|  | rs32074 | 5 | 156111450 | 11600 | 0.2811 |
|  | rs6556717 | 5 | 156002438 | 11608 | 0.2813 |
|  | rs1422220 | 5 | 156060067 | 11612 | 0.2815 |
|  | rs7447475 | 5 | 155779168 | 12509 | 0.3131 |
|  | rs11135380 | 5 | 156182048 | 12939 | 0.3284 |
|  | rs11743060 | 5 | 155784910 | 13651 | 0.3531 |
|  | rs172240 | 5 | 156166739 | 13702 | 0.3548 |
|  | rs32080 | 5 | 156103226 | 13907 | 0.3616 |
|  | rs140615 | 5 | 155811050 | 14081 | 0.3687 |
|  | rs6883722 | 5 | 155875529 | 14927 | 0.3976 |
|  | rs1875963 | 5 | 155803661 | 16068 | 0.4368 |
|  | rs2055609 | 5 | 155804271 | 16504 | 0.4518 |
|  | rs2652324 | 5 | 156153513 | 16885 | 0.4649 |
|  | rs13184996 | 5 | 155800694 | 17111 | 0.4731 |
|  | rs2135028 | 5 | 155826540 | 17452 | 0.4846 |
|  | rs1368300 | 5 | 155922099 | 17916 | 0.5005 |
|  | rs157341 | 5 | 156133899 | 18740 | 0.5308 |
|  | rs2135038 | 5 | 155766979 | 19103 | 0.5436 |
|  | rs1504926 | 5 | 155796753 | 19463 | 0.5577 |
|  | rs7715464 | 5 | 155809052 | 19777 | 0.5690 |
|  | rs256823 | 5 | 155932998 | 19778 | 0.5690 |
|  | rs39926 | 5 | 156116412 | 19868 | 0.5724 |
|  | rs282466 | 5 | 156153399 | 19964 | 0.5758 |
|  | rs42685 | 5 | 156065196 | 20860 | 0.6097 |
|  | rs11135381 | 5 | 156182200 | 21103 | 0.6182 |
|  | rs6862189 | 5 | 155819278 | 21319 | 0.6267 |
|  | rs281046 | 5 | 156082997 | 23195 | 0.6965 |
|  | rs157335 | 5 | 156117447 | 23775 | 0.7185 |
|  | rs10515745 | 5 | 156170837 | 23803 | 0.7196 |
|  | rs11955986 | 5 | 156168617 | 24564 | 0.7496 |
|  | rs12521174 | 5 | 156141605 | 24594 | 0.7515 |
|  | rs7715923 | 5 | 155957439 | 24672 | 0.7539 |
|  | rs11744039 | 5 | 156103611 | 24703 | 0.7553 |
|  | rs905788 | 5 | 155862225 | 24864 | 0.7619 |
|  | rs280475 | 5 | 156015955 | 26133 | 0.8125 |
|  | rs3797574 | 5 | 156017085 | 28021 | 0.8853 |
|  | rs12652669 | 5 | 155876204 | 28512 | 0.9035 |
|  | rs32063 | 5 | 156065593 | 28706 | 0.9111 |
|  | rs17053613 | 5 | 155971322 | 29113 | 0.9260 |
|  | rs7725121 | 5 | 155753983 | 29468 | 0.9389 |
|  | rs10059872 | 5 | 155779927 | 30182 | 0.9667 |
|  | rs10491465 | 5 | 156080965 | 30794 | 0.9909 |
|  | rs10062636 | 5 | 155804588 | 30905 | 0.9950 |
|  |  |  |  |  |  |
| *TAZ* | rs1130929 | X | 153633359 | 7734 | 0.1633 |
|  | rs4898494 | X | 153654122 | 8103 | 0.1751 |
|  | rs2283762 | X | 153632196 | 11957 | 0.2936 |
|  | rs11156577 | X | 153660041 | 14107 | 0.3694 |
|  |  |  |  |  |  |
| *TCAP* | rs2271308 | 17 | 37817482 | 15502 | 0.4175 |
|  | rs903502 | 17 | 37829604 | 21618 | 0.6370 |
|  | rs1877031 | 17 | 37814080 | 25266 | 0.7778 |
|  | rs11869286 | 17 | 37813856 | 25823 | 0.8014 |
|  | rs881844 | 17 | 37810218 | 26469 | 0.8270 |
|  |  |  |  |  |  |
| *TMPO* | rs12580397 | 12 | 98932090 | 2022 | 0.0260 |
|  | rs4394881 | 12 | 98912028 | 3489 | 0.0567 |
|  | rs7135956 | 12 | 98921343 | 13689 | 0.3543 |
|  | rs11768 | 12 | 98908812 | 18775 | 0.5320 |
|  | rs1439775 | 12 | 98907379 | 19867 | 0.5723 |
|  | rs11109522 | 12 | 98930964 | 21507 | 0.6329 |
|  | rs17459334 | 12 | 98927830 | 22846 | 0.6838 |
|  | rs2068060 | 12 | 98939120 | 26130 | 0.8123 |
|  |  |  |  |  |  |
| *TNNC1* | rs1541495 | 3 | 52492707 | 4484 | 0.0794 |
|  | rs634382 | 3 | 52482851 | 8224 | 0.1792 |
|  | rs9855470 | 3 | 52493275 | 8763 | 0.1947 |
|  | rs10865971 | 3 | 52481406 | 22310 | 0.6626 |
|  |  |  |  |  |  |
| *TNNI3* | rs3729711 | 19 | 55667647 | 2060 | 0.0266 |
|  | rs3729709 | 19 | 55667806 | 15909 | 0.4310 |
|  | rs2288529 | 19 | 55665689 | 16947 | 0.4666 |
|  | rs16986278 | 19 | 55656149 | 20247 | 0.5868 |
|  | rs7255625 | 19 | 55678688 | 20366 | 0.5915 |
|  | rs3826890 | 19 | 55661907 | 21070 | 0.6171 |
|  | rs3848617 | 19 | 55673245 | 21757 | 0.6426 |
|  | rs3729841 | 19 | 55665410 | 22346 | 0.6639 |
|  | rs3729838 | 19 | 55668310 | 25746 | 0.7980 |
|  | rs2434454 | 19 | 55657041 | 25767 | 0.7988 |
|  |  |  |  |  |  |
| *TNNT2* | rs2275860 | 1 | 201333703 | 887 | 0.0081 |
|  | rs11807637 | 1 | 201331335 | 930 | 0.0087 |
|  | rs3020556 | 1 | 201343115 | 3698 | 0.0613 |
|  | rs16848494 | 1 | 201350983 | 6364 | 0.1247 |
|  | rs1892028 | 1 | 201336641 | 9440 | 0.2157 |
|  | rs868407 | 1 | 201341341 | 10157 | 0.2379 |
|  | rs4915232 | 1 | 201347946 | 10934 | 0.2620 |
|  | rs3730238 | 1 | 201330429 | 15716 | 0.4249 |
|  | rs2799691 | 1 | 201348343 | 19187 | 0.5464 |
|  | rs3729845 | 1 | 201334795 | 26312 | 0.8205 |
|  | rs16848462 | 1 | 201338107 | 30209 | 0.9679 |
|  | rs2799686 | 1 | 201347315 | 30672 | 0.9862 |
|  |  |  |  |  |  |
| *TPM1* | rs11071721 | 15 | 63350218 | 356 | 0.0018 |
|  | rs3803499 | 15 | 63343318 | 2327 | 0.0318 |
|  | rs4775609 | 15 | 63332009 | 4878 | 0.0888 |
|  | rs1071646 | 15 | 63351840 | 10630 | 0.2528 |
|  | rs8042050 | 15 | 63355871 | 11004 | 0.2642 |
|  | rs3803501 | 15 | 63352092 | 13211 | 0.3378 |
|  | rs4774471 | 15 | 63332645 | 20996 | 0.6143 |
|  | rs4075583 | 15 | 63340227 | 25847 | 0.8021 |
|  | rs17752921 | 15 | 63343631 | 27581 | 0.8696 |
|  | rs1972041 | 15 | 63360847 | 30220 | 0.9682 |
|  |  |  |  |  |  |
| *TTN* | rs1017323 | 2 | 179488736 | 2733 | 0.0404 |
|  | rs2291306 | 2 | 179638238 | 2899 | 0.0436 |
|  | rs35813871 | 2 | 179650408 | 4208 | 0.0728 |
|  | rs16866378 | 2 | 179393111 | 4327 | 0.0756 |
|  | rs4894030 | 2 | 179461221 | 11438 | 0.2768 |
|  | rs3816849 | 2 | 179667090 | 12340 | 0.3068 |
|  | rs12463674 | 2 | 179432185 | 14370 | 0.3790 |
|  | rs6715901 | 2 | 179650954 | 14415 | 0.3805 |
|  | rs16866420 | 2 | 179491235 | 14623 | 0.3868 |
|  | rs2291310 | 2 | 179623758 | 15049 | 0.4021 |
|  | rs12464787 | 2 | 179440029 | 15660 | 0.4232 |
|  | rs2291311 | 2 | 179629461 | 17313 | 0.4797 |
|  | rs7590037 | 2 | 179499038 | 18324 | 0.5151 |
|  | rs2306636 | 2 | 179634936 | 18341 | 0.5158 |
|  | rs3769858 | 2 | 179667806 | 18420 | 0.5189 |
|  | rs2129108 | 2 | 179674929 | 18762 | 0.5316 |
|  | rs7585334 | 2 | 179620951 | 18997 | 0.5395 |
|  | rs10164753 | 2 | 179438866 | 19278 | 0.5504 |
|  | rs12476289 | 2 | 179641975 | 20318 | 0.5901 |
|  | rs2562838 | 2 | 179579212 | 23781 | 0.7188 |
|  | rs746578 | 2 | 179605180 | 24859 | 0.7616 |
|  | rs12614435 | 2 | 179489726 | 26446 | 0.8262 |
|  | rs4894048 | 2 | 179638721 | 26780 | 0.8393 |
|  | rs10497522 | 2 | 179652047 | 27959 | 0.8828 |
|  | rs1484118 | 2 | 179619958 | 28126 | 0.8891 |
|  | rs4893853 | 2 | 179632496 | 30171 | 0.9661 |
|  | rs890578 | 2 | 179414633 | 30237 | 0.9690 |
|  | rs10497520 | 2 | 179644855 | 30784 | 0.9906 |
|  |  |  |  |  |  |
| *VCL* | rs1403629 | 10 | 75773362 | 8044 | 0.1731 |
|  | rs2279648 | 10 | 75867193 | 9359 | 0.2127 |
|  | rs2395075 | 10 | 75768180 | 10371 | 0.2449 |
|  | rs11000868 | 10 | 75841498 | 11025 | 0.2648 |
|  | rs11000864 | 10 | 75818146 | 11720 | 0.2856 |
|  | rs10824071 | 10 | 75873639 | 14559 | 0.3849 |
|  | rs10458640 | 10 | 75780838 | 17102 | 0.4728 |
|  | rs3793921 | 10 | 75868114 | 19083 | 0.5431 |
|  | rs2131961 | 10 | 75841456 | 19196 | 0.5469 |
|  | rs10762575 | 10 | 75811243 | 21333 | 0.6271 |
|  | rs4746172 | 10 | 75855842 | 22888 | 0.6852 |
|  | rs11814533 | 10 | 75795561 | 23680 | 0.7156 |
|  | rs4746166 | 10 | 75778687 | 29776 | 0.9508 |

* Ranking acording to all analyzed markers (30,920) using logistic regression adjusted for gender
