# Supplementary material for: Genetic Association Study Identifies HSPB7 as a Risk Gene for Idiopathic Dilated Cardiomyopathy
Source: PLoS Genet. 2010 Oct 21;6(10):e1001167. doi: 10.1371/journal.pgen.1001167 (PMC2958814; doi:10.1371/journal.pgen.1001167)
Supplement: Table S3 — Primer sequences for PCR amplification of the HSPB7 coding region and product sizes. (0.03 MB DOC) [file pgen.1001167.s003.doc]

Table S3. Primer sequences for PCR amplification of the *HSPB7* coding region and product sizes

| Amplicon | Forward (5'-3') | Reverse (5'-3') | Size (bp) |
| --- | --- | --- | --- |
| Exon 1-coding | ctggaatgtcaggctgtgag | agggccacaactgttcctta | 675 |
| Exon 2 | gtgggctgtaggaatgagga | agttagccctgggggagtt | 426 |
| Exon 3-coding | ggggttagaatggggagaag | ccctagtttgggaggatggt | 419 |
